# Clinical correlates of arterial lactate levels in patients with ST-segment elevation myocardial infarction at admission: a descriptive study

**DOI:** 10.1186/cc9253

**Published:** 2010-09-09

**Authors:** Robert P Vermeulen, Miriam Hoekstra, Maarten WN Nijsten, Iwan C van der Horst, L Joost van Pelt, Gillian A Jessurun, Tiny Jaarsma, Felix Zijlstra, Ad F van den Heuvel

**Affiliations:** 1 Department of Cardiology, Thorax Center, University Medical Center Groningen, University of Groningen, Hanzeplein 1 Groningen, The Netherlands; 2 Department of Critical Care, University Medical Center Groningen, University of Groningen, Hanzeplein 1 Groningen, The Netherlands; 3 Department of Laboratory Medicine, University Medical Center Groningen, University of Groningen, Hanzeplein 1 Groningen, The Netherlands; 4 Department of Cardiology, Scheper Ziekenhuis, Boermarkeweg 60 Emmen, The Netherlands; 5 Department of Cardiology, Erasmus Medical Center, 's-Gravendijkwal 230 Rotterdam, The Netherlands

## Abstract

**Introduction:**

Blood lactate measurements can be used as an indicator of hemodynamic impairment and relate to mortality in various forms of shock. Little is known at the moment concerning the clinical correlates of systemic lactate in patients with ST-segment elevation myocardial infarction (STEMI).

**Methods:**

To assess the relation of systemic arterial lactate levels in STEMI patients with clinical correlates at presentation in the catheterization laboratory, we measured arterial lactate levels with a rapid point-of-care technique, immediately following femoral sheath insertion. The study population (n= 1,176) was divided into tertiles with lactate levels ≤1.1 (n = 410), 1.2 to 1.7 (n = 398) and ≥1.8 mmol/l (n = 368). We compared both baseline characteristics and outcome measures of the three lactate groups.

**Results:**

Factors independently associated with higher lactate levels were hypotension, heart rate, thrombolysis in myocardial infarction (TIMI) flow 0 to 1, diabetes and non-smoking. Mortality at 30 days in the three groups was 2.0%, 1.5% and 6.5%. The latter group also showed lower blush grades and greater enzymatic infarct sizes. An intra aortic balloon pump (IABP) was used more frequently in patients with higher lactate levels (4.2%, 7.6% and 14.7%).

**Conclusions:**

In STEMI patients, impaired hemodynamics, worse TIMI flow and non-smoking were related to increased arterial lactate levels. Higher lactate levels were independently related with 30-day mortality and an overall worse response to percutaneous coronary intervention (PCI). In particular, acute mortality was related to admission lactates ≥1.8 mmol/L. Point-of-care measurement of arterial lactate at admission in patients with STEMI has the potential to improve acute risk stratification.

## Introduction

The clinical value of circulating lactate has been extensively demonstrated in critical care medicine [1]. Blood lactate measurements can be used as an indicator of hemodynamic impairment and as a predictor of outcome in various forms of shock. In patients with cardiogenic shock, several studies document marked elevations in circulating lactate [2-9]. One of the most frequent causes of circulatory shock is acute myocardial infarction. When tissue perfusion is impaired during acute myocardial infarction, decreased oxygen delivery can induce muscle cells to preferentially use glycolysis and produce lactate from pyruvate, rather than oxidize pyruvate for mitochondrial energy production. In patients with ischemic heart disease, the amount of lactate released by the myocardium has been shown to be related to the severity of coronary artery disease [10]. For patients with myocardial infarction, circulating venous lactate levels have been shown to be increased [11]. However, long transport times between blood sampling and analysis may have led to falsely high results [12]. Today rapid point-of-care analyzers are generally available, enabling fast measurement of lactate levels.

The aim of this study was to determine the clinical correlates of systemic arterial lactate levels in patients with ST-elevation myocardial infarction (STEMI) prior to primary percutaneous coronary intervention (PCI) using a rapid point-of-care analyzer. We investigated whether hemodynamic parameters and other patient characteristics at admission were related to lactate levels. In addition, we related lactate levels at presentation to 30-day mortality, and other outcome measures such as TIMI-flow (thrombolysis in myocardial infarction), peak cardiac enzymes, ST-segment elevation resolution and myocardial blush grade (MBG).

## Materials and methods

### Population

This was a prospective, observational study performed in the catheterization laboratory of the University Medical Center Groningen, The Netherlands. In The Netherlands, primary PCI is the standard treatment for all patients with STEMI. All patients with diagnosed STEMI within the region of a hospital with interventional capacities covered by a 90-minute radius by ambulance are either transported directly to the catheterization laboratory or referred by other non-interventional centers. Direct transport for mechanical reperfusion therapy has repeatedly been proven to improve survival in STEMI patients [13,14]. Before primary PCI, patients receive as premedication 5,000 IU of heparin, 900 mg acetyl salicylic acid and 600 mg clopidogrel, either in the ambulance, at presentation in the catheterization laboratory or in the referring hospital.

We evaluated all patients who were diagnosed with STEMI and treated with primary PCI in the catheterization laboratory of our hospital from January 2006 to September 2008. STEMI was diagnosed in patients with symptoms suggesting acute myocardial ischemia lasting more than 30 minutes, with the onset of symptoms less than 12 hours previously, and with ST-segment elevation of more than 0.1 mV in two or more leads on the ECG. Patients intubated prior to primary PCI after cardiopulmonary resuscitation were excluded, because lactate levels have been shown to be considerably elevated in resuscitated patients [15,16]. The institutional review board exempted the study from formal review.

### Methods of measurement

To measure lactate levels an arterial blood sample was taken directly after femoral sheath insertion using a self-filling 1.5 ml PICO70 arterial sampler containing 60 IU of lithium heparin (Radiometer, Copenhagen, Denmark). Lactate was measured within three to four minutes on an ABL 700/800 series analyzer (Radiometer) with a lower detection limit of 0.1 mmol/L. In a whole blood sample, the ABL analyzer converts lactate to pyruvate and hydrogen peroxyde. The hydrogen peroxyde is then reduced on a platinum anode, inducing a current proportional to the lactate level in the sample. This method has been shown to be fast and accurate, with results comparable to those from central laboratory analyzers [17,18]. During the procedure, the operator was not informed of the result of the lactate measurement.

Patient characteristics and details about the procedure and location of the infarction were taken from medical charts and the database of the catheterization laboratory. Blood pressure was measured in the ascending aorta. Hypotension was defined as a systolic blood pressure of ≤90 mmHg [19]. The primary end point was death from all causes at 30 days. ST-segment elevation resolution was determined by comparing the 12-lead electrocardiogram (ECG) obtained directly prior to PCI to an ECG made 30 to 60 minutes after PCI. The percentages of resolution of the ST-segment elevation were classified into three categories: >70%, 30 to 70% and <30%. MBG after PCI was assessed as previously described [20], as well as the maximum levels of creatine kinase (CK) [21] and troponine T. As liver dysfunction can lead to impaired lactate clearance, all patients were screened for laboratory signs of liver dysfunction. We also specifically looked at metformin use in diabetic patients as it is known that metformin can lead to elevated lactate levels. Coronary blood flow before and after primary PCI was classified using the TIMI-flow grades. In the description of the results, MBG and TIMI-flow were dichotomized as 0 to 1 or 2 to 3. The use of IABP (intra aortic balloon pump) was also recorded.

### Statistical analysis

All data were analyzed using SPSS version 14 (SPSS, Chicago, IL, USA). The study population was divided into tertiles based on admission lactate level. To indicate variation, we used means ± SD for normal distributions or medians with interquartile ranges (Q25 to Q75) for skewed distributions. Where appropriate, ANOVA, Kruskal-Wallis H, Chi-square tests and ordinal regression tests were used to make comparisons among the three groups of patients. To calculate multivariate relations of lactate and other baseline characteristics that were available before the first angiogram to 30-day mortality, we used binary logistic regression analysis. Lactate was entered into the analysis as a continuous variable after lognormal transformation. The variables of age, systolic blood pressure, heart rate, anterior infarction, diabetes, smoking and lactate were entered in a single step. To calculate differences in Kaplan-Meier curves we performed a logrank test (Mantel-Cox.). A *P*-value of ≤.05 was considered statistically significant.

## Results

### Baseline clinical characteristics and lactate levels

From January 2006 to September 2008, 1,420 patients with STEMI were treated with PCI at the University Medical Center of Groningen. Of these patients 73 were intubated (5%). For the 73 intubated patients, the median lactate level was 4.2 (1.5 to 7.9) with a range of 0.7 to 18.0 mmol/L, and mortality at 30 days was 40%.

In 13% of STEMI patients, an arterial lactate measurement was not performed prior to the primary PCI. Logistical reasons as technical maintenance of the ABL analyzer or the analyzer being in calibration mode were the cause of most of the missing cases. With regard to baseline characteristics and 30-day mortality, missing patients were similar to included patients. A total of 1,176 patients were included in the subsequent analyses.

Lactate levels were positively skewed, with a mean level of 1.65 ± 1.01 mmol/L and a median level of 1.4 (1.0 to 2.0), range 0.5 to 10.9. Of the 1,176 included patients, most were male (72%) and the mean age was 64 ± 13. Anterior infarction was present in 39% of patients. Mean systolic blood pressure was 127 ± 27 mmHg, with 6% of patients presenting with hypotension. The mean heart rate was 78 ± 19 beats per minute. TIMI-flow before PCI was rated as 0/1 in 66% of patients. Angina was present in the days to weeks before infarction in 50% of patients. In only three patients liver dysfunction was observed. The study population was divided into tertiles with lactate levels ≤1.1 (n= 410), 1.2 to 1.7 (n = 398) and ≥1.8 mmol/L (n = 368).

Table 1 displays the baseline characteristics for the three lactate groups. In univariate analysis, patients in the three groups were different with respect to heart rate, incidence of hypotension and poor TIMI-flow, history of diabetes and history of smoking and admission creatinine. After regression analysis, clinical characteristics independently related to higher lactate levels were hypotension (*P *< .001), higher heart rate (*P *= .002), TIMI-flow 0 to 1 (*P *< .001), diabetes (*P *= .001) and non-smoking (*P *< .001). Of 128 diabetic patients, 47 used metformin (37%). Lactate levels were higher in diabetic patients who used metformin compared to other diabetic patients: 2.3 mmol/L vs 1.7 mmol/L (*P *= .005).

**Table 1:** Baseline characteristics, by tertile of lactate level

	Lac ≤ 1.1 mmol/L n= 410	Lac 1.2-1.7 mmol/L n= 398	Lac ≥ 1.8 mmol/L n= 368	*P*
Gender (male)	73.9	74.4	72.0	.738
Age	63 ± 13	63 ± 13	64 ± 12	.160
BMI	26 ± 4	27 ± 13	28 ± 5	.133
Hypotension	4.7	4.0	14.3	< .001 *
Heart rate	77 ± 16	78 ± 19	81 ± 21	.007 *
Anterior infarction	40.9	36.0	40.9	.273
Time in minutes from symptom onset to lactate measurement	176 (128 to 297)	160 (110 to 305)	173 (123 to 272)	.217
TIMI-flow 0 to 1	56.7	71.3	69.5	< .001 *
Pre-infarct angina	53.8	48.4	46.7	.134
Risk factors:				
Hypertension	31.7	38.2	41.3	.024
Diabetes	7.4	8.9	19.0	< .001 *
Hypercholesterolemia	32.5	37.0	31.5	.321
Smoking	56.5	47.4	40.2	< .001 *
Family History	42.9	45.2	46.6	.607
Previous myocardial infarction	9.7	11.2	12.0	.585
Previous PCI	8.6	7.2	9.0	.653
Previous CABG	2.5	2.4	3.2	.789
Admission Hemoglobin mmol/L	8.0 ± 1.1	8.2 ± 1.1	8.2 ± 1.2	.217
Creatinine μmol/L	77 (66 to 91)	80 (68 to 93)	83 (70 to 99)	.002

*Independent correlates to admission lactate after regression analysis. Numbers as %, mean ± SD or median (Q25 to Q75); CABG, coronary artery bypass grafting; PCI, percutaneous coronary intervention; TIMI, thrombolysis in myocardial infarction.

### Lactate levels and outcome measures

**Figure 1:**
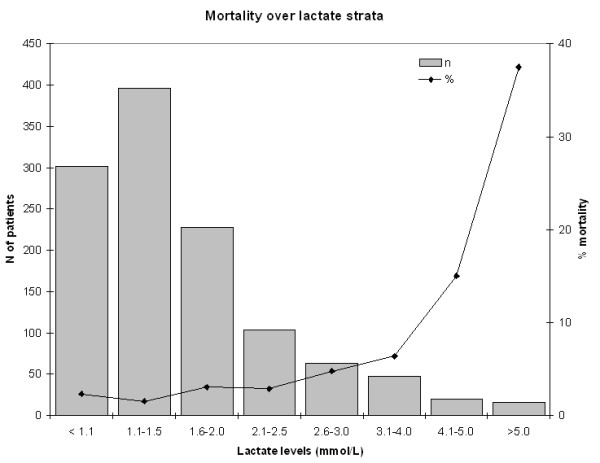
**Distribution of 30-day mortality in relation to admission lactates**.

At 30 days, overall mortality was 3.2% (n = 38). In-hospital mortality was 2.2% (n = 26). The distribution of 30-day mortality in relation to admission lactates is displayed in Figure 1. For patients with lactate levels >4.0 mmol/L mortality showed a marked increase. The relations of the lactate tertiles with outcome measures are displayed in Table 2. In the group with lactates ≥1.8 mmol/L mortality was increased (2.0 vs. 1.5 vs. 6.5%, *P *< .001). In addition, for all other outcome measures except TIMI-flow and percentage of ST-segment resolution a significant difference between the three groups was found. When comparing patients in the first tertile to those in the second tertile separately, we found that the incidence of MBG 2/3 was lower in the second tertile, and peak cardiac enzymes levels were higher. These outcome measures were similar when comparing patients from the first tertile to patients from the last tertile. IABP was needed in 4.2%, 7.6% and 14.7% of patients in each tertile (*P *< .001).

**Table 2:** Outcome parameters by tertile of lactate level

	Lac ≤ 1.1 mmol/L (n = 410)	Lac 1.2 to 1.7 mmol/L (n = 98)	Lac ≥ 1.8 mmol/L (n = 68)	*P*
30-day mortality	2.0	1.5	6.5	< .001
TIMI-flow 2 to 3	96.5	97.2	93.9	.055
Myocardial blush grade 2 to 3	82.0	76.8	67.2	< .001
ST-segment elevation resolution after PCI				
>70%	64.4	59.0	54.5	.120
30 to 70%	23.5	24.2	28.2	
< 30%	12.1	16.8	17.3	
CK total (peak, units/L)	789 (239 to 787)	1,175(542 to 2,239)	1,296(488 to 2,801)	< .001
Troponine T (peak, μg/L)	2.13 (0.54 to 5.90)	3.63(1.45 to 7.11)	3.89(1.41 to 8.02)	< .001

Numbers as %, mean ± SD or median (Q25 to Q75). CK, creatine kinase; PCI, percutaneous coronary intervention; TIMI, thrombolysis in myocardial infarction.

**Figure 2:**
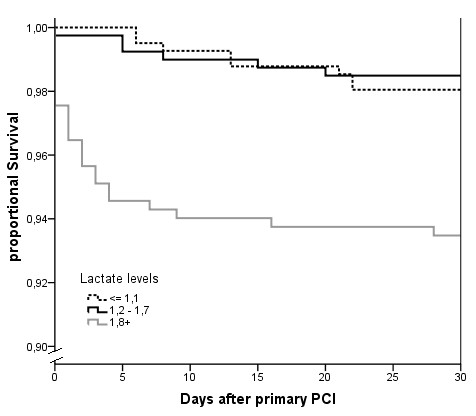
**Kaplan-Meier curve displaying 30-day proportional survival after primary PCI. **PCI, percutaneous coronary intervention.

The Kaplan-Meier curve in Figure 2 displays 30-day proportional survival after primary PCI, stratified by the three tertiles of admission lactate. The differences in survival were significant (*P *< .001). We further examined the survival time of the non-survivors of the first 30 days. In the non-survivors from the first group, median survival in days was 13 (7 to 22), whereas for non-survivors from the second group median survival was 7 (4 to 16). For non-survivors from the third group, median survival was one day (0 to 4) (*P *< .001).

In multivariate analysis, we determined if clinical characteristics and risk factors readily available at presentation (age, systolic blood pressure, heart rate, anterior infarction, diabetes, smoking and admission lactate) were independently related to 30-day mortality. Table 3 shows the strength of each independent relation and the level of significance. Lactate was independently related to 30-day mortality, together with age, systolic blood pressure, heart rate and smoking status. Similar results except for smoking status were observed after excluding diabetic patients from the multivariate analysis.

**Table 3:** Multivariate relations of clinical characteristics to 30-day mortality

Baseline characteristic	Hazard Ratio	95% CI	*P*
Age	1.10	1.16 to 1.65	< .001
Systolic blood pressure	0.96	0.95 to 0.98	< .001
Heart rate	1.02	1.00 to 1.04	.028
Anterior infarction	0.56	0.24 to 1.29	.174
Diabetes	0.64	0.22 to 1.89	.416
Smoking	0.31	0.12 to 0.82	.017
Lactate	2.52	1.12 to 5.66	.025

CI, confidence interval.

## Discussion

In this study we found that elevated arterial lactate levels measured at presentation in the catheterization laboratory prior to PCI were related to hypotension, higher heart rate, poor TIMI-flow, diabetes and non-smoking. Moreover, increased lactate levels were associated with worse outcome measures including increased 30-day mortality, lower MBG, larger enzymatic infarct size and increased use of IABP. Fifty percent of the non-survivors with admission lactates ≥1.8 mmol/L died within a day after PCI.

The additional value of an early lactate measurement with regard to patient risk stratification is probably most pronounced in patients with strongly elevated lactate levels, as has been demonstrated in patients with sepsis [22]. The sharp increase in mortality for patients with admission lactates >4.0 mmol/L as seen in Figure 1 suggests that in these patients intensified medical care, such as IABP, may be considered, even after a successful PCI. The analysis of survival times for the non-survivors corroborates this observation. That admission lactate had the strongest independent relation to mortality in a model with other easily obtainable clinical parameters as age, hypotension and heart rate, adds to our belief that lactate measurement could be a part of standard laboratory assessment of patients with STEMI at admission in the hospital.

The finding that mildly elevated lactate levels were associated with non-smoking, may be a reflection of the smoker's paradox [23,24]. Although age was not associated with elevated lactate levels, we too observed that smokers suffered from STEMI at a younger age than non-smokers (58 vs. 69 years, *P *< .001).

It was already recognized in 1961 that circulating lactate provides information about the hemodynamic condition of a patient [25,26] and that lactate was strongly related to survival in 142 patients with shock, including 40 patients suffering from cardiogenic shock [5]. Many studies in the emergency department and in the intensive care unit have since demonstrated that increased lactate levels are a strong predictor of early and late mortality in patients with cardiogenic shock, traumatic shock, sepsis and septic shock or liver failure [27-29], as in non-diabetic patients with STEMI [30]. In this last study, in 253 patients lactate was measured at the CCU after PCI. Lactate, homeostatic model assessment index (HOMA) and C-peptide were found to be independent predictors of mortality in the CCU. Lactate was also found to be higher in patients with increased insulin resistance. These results indicate that also in our study increased insulin resistance may have been causal in both the increased lactates and the adverse outcome, even in non-diabetics.

Another study that measured venous lactate levels in patients with myocardial infarction found that two hours after symptom onset, most patients had elevated lactate levels [11]. Out of 64 patients with myocardial infarction, 59 (92%) had venous lactate levels ≥1.5 mmol/L when measured in a central laboratory. Our findings are in contrast with these results. Apart from using arterial blood, another difference is that in our study, all lactate measurements were performed within three to four minutes from sampling. During transport, lactate levels may rise 0.1 to 1.2 mmol/L in the first hour, depending on the circumstances [12]. Prompt measurement on a point-of-care analyzer in the catheterization laboratory probably resulted in more accurate and lower lactate levels compared to lactate measurement in a central laboratory. The previous studies also claimed on the basis of a few patients a high negative predictive value of venous lactate for acute myocardial infarction. In a much larger patient group, we found that normal lactate levels occur in half of the patients with confirmed STEMI.

These observations are in line with established physiological principles. Lactate through pyruvate provides under normal circumstances 10 to 40% of the myocardial fuel supply, making the heart an important net consumer of lactate. Impaired oxygen supply to parts of the myocardium can turn the heart into a lactate producer instead of a lactate consumer, as has been observed in several models [10,31]. Hypotension, stress and glycometabolic dysregulation [30] may also lead to increased lactate levels. Our findings suggest further avenues of interest. One single lactate measurement can provide insight into the hemodynamic condition of the patient, but the fact that patients with high patency rates have lower lactate levels, could mean that after successful primary PCI, lactate levels normalize. Nowadays laboratory equipment that promptly performs lactate measurements is available to study the time course of lactate levels in patients with STEMI.

Our study has a number of limitations. First, this study was performed in a single center. Second, not all STEMI patients were included: in 13% of patients, a lactate measurement at presentation was not performed. However, baseline characteristics and mortality figures of missing patients were similar to included patients; therefore, it is unlikely that our results were greatly influenced by selection bias. Additional parameters such as glucose, glycosylated hemoglobin (HbA1c) and brain natriuretic peptide (NT-pro BNP) that may be related with lactate levels were not available.

## Conclusions

At admission, increasing arterial lactate levels were associated with higher heart rate, hypotension, poor TIMI-flow, diabetes and non-smoking. Higher lactate levels were related to worse PCI outcome measures and increased 30-day mortality. Rapid point-of-care measurement of arterial lactate at presentation in patients with STEMI has the potential to improve acute risk stratification.

## Key messages

• Point-of-care analysis enables fast and accurate measurement of arterial lactate levels at the catheterization laboratory.

• Lactate levels in STEMI patients at the catheterization laboratory were related to hemodynamics and TIMI flow.

• Increased lactate levels in STEMI patients were associated with 30-day mortality.

• Half of the non-survivors with admission lactate levels of ≥1.8 mmol/L died within a day after presentation in the hospital.

## Abbreviations

CABG: coronary artery bypass grafting; CK: creatine kinase; ECG: electrocardiogram; HOMA: homeostatic model assessment index; IABP: intra aortic balloon pump; MBG: myocardial blush grades; PCI: percutaneous coronary intervention; SD: standard deviation; STEMI: ST-segment elevation myocardial infarction; TIMI: thrombolysis in myocardial infarction.

## Competing interests

The authors declare that they have no competing interests.

## Authors' contributions

RV collected data, analyzed and interpreted the data, and drafted the manuscript. MH helped with the analyses and corrected the manuscript drafts. MN and FZ conceived the study, analyzed and interpreted the data, and drafted and corrected the manuscript. IH and TJ helped with interpreting the data and corrected the manuscript drafts. GJ, AH and JP assisted in data collection and corrected manuscript drafts. RV, MN and FZ are responsible for the paper as a whole.
